# Antidepressant Drugs and Physical Activity: A Possible Synergism in the Treatment of Major Depression?

**DOI:** 10.3389/fpsyg.2020.00857

**Published:** 2020-05-06

**Authors:** Claudia Savia Guerrera, Giovanna Furneri, Margherita Grasso, Giuseppe Caruso, Sabrina Castellano, Filippo Drago, Santo Di Nuovo, Filippo Caraci

**Affiliations:** ^1^Department of Biomedical and Biotechnological Sciences, University of Catania, Catania, Italy; ^2^Department of Educational Sciences, University of Catania, Catania, Italy; ^3^Department of Laboratories, Oasi Research Institute – IRCCS, Troina, Italy; ^4^Department of Drug Sciences, University of Catania, Catania, Italy

**Keywords:** depression, physical activity, stress, affective symptoms, cognition, brain-derived neurotrophic factor, transforming-growth-factor-β1

## Abstract

Major depressive disorder (MDD) is a severe mental illness that affects 5–20% of the general population. Current antidepressant drugs exert only a partial clinical efficacy because approximately 30% of depressed patients failed to respond to these drugs and antidepressants produce remission only in 30% of patients. This can be explained by the fact that the complex pathophysiology of depression has not been completely elucidated, and treatments have been mainly developed following the “monoaminergic hypothesis” of depression without considering the key role of other factors involved in the pathogenesis of MDD, such as the role of chronic stress and neuroinflammation. Chronic stress acts as a risk factor for the development of MDD through the impairment of neurotrophins signaling such as brain-derived neurotrophic factor (BDNF) and transforming-growth-factor-β1 (TGF-β1). Stress-induced depressive pathology contributes to altered BDNF level and function in MDD patients and, thereby, an impairment of neuroplasticity at the regional and circuit level. Recent studies demonstrate that aerobic exercise strongly increases BDNF production and it may contribute as a non-pharmacological strategy to improve the treatment of cognitive and affective symptoms in MDD. Here we will provide a general overview on the possible synergism between physical activity and antidepressants in MDD. Physical activity can synergize with antidepressant treatment by rescuing neurotrophins signaling in MDD patients, promoting neuronal health and recovery of function in MDD-related circuits, finally enhancing pharmacotherapeutic response. This synergism might be particularly relevant in elderly patients with late-life depression, a clinical subgroup with an increased risk to develop dementia.

## Introduction

Major depressive disorder (MDD) is a severe and a common mental illness affecting more than 264 million people worldwide (Gbd 2017 Disease and Injury Incidence and Prevalence Collaborators, 2018). The World Health Organization (WHO) describes depression, also indicated as MDD or clinical depression, as a mental disorder characterized by sleep and appetite disturbances, variation of mood, loss of energy, and psychomotor retardation^1^.

Among the different hypotheses that have been proposed to explain MDD pathophysiology, the “monoaminergic hypothesis” has been initially validated with the development of monoaminergic antidepressants. Based on this hypothesis an impairment of monoaminergic systems [serotonin (5-HT), noradrenaline, and dopamine] has been considered a primary event for the onset of affective and cognitive symptoms in MDD (Hirschfeld, 2000; Hamon and Blier, 2013). Therefore, the majority of antidepressant drugs have been developed according to this hypothesis, representing a useful therapeutic tool (Lopez-Munoz and Alamo, 2009); unfortunately around 30% of depressed patients are considered treatment resistant (Caraci et al., 2018a), probably because emerging additional factors involved in the pathophysiology of MDD, such as chronic stress and neuroinflammation, should be considered (Caraci et al., 2018b). The pathological effects of stress on hippocampus have contributed to the development of the so-called “neurotrophic hypothesis” according to which neurotrophic factors play a key role in the etiology of depression (Altar, 1999; Duman and Li, 2012; Jaggar et al., 2019). This hypothesis suggests that depression derives from decreased neurotrophic support resulting in neuronal atrophy, decreased hippocampal neurogenesis, and loss of glial cells (Duman and Monteggia, 2006). A hyperactivation of the hypothalamic-pituitary-adrenal (HPA) axis has been found in the 50% of depressed patients (Krishnan and Nestler, 2008) and several evidences identify chronic stress, linked to an impairment of neurotrophins such as brain-derived neurotrophic factor (BDNF) and transforming-growth-factor-β1 (TGF-β1) (Caraci et al., 2018b), as a risk factor for the development of MDD (Pittenger and Duman, 2008; Caraci et al., 2010). A significant decrease of BDNF levels has been demonstrated in animal models of depression stress-induced (Berry et al., 2012) as well as in depressed patients (Angelucci et al., 2005). Likewise, a decrease of TGF-β1 levels has been observed in hippocampus and cortex of animal models of depression (Yu et al., 2011); furthermore, several studies carried out in depressed patients have demonstrated that plasma TGF-β1 levels are reduced and correlate with depression severity (Myint et al., 2005; Rush et al., 2016). A chronic treatment with first- and second-generation antidepressants rescues BDNF levels in different preclinical models of depression (Duman and Monteggia, 2006), while selective serotonin reuptake inhibitors (SSRIs) drugs and the new multimodal antidepressant vortioxetine are able to reverse the depressive-like phenotype and memory deficits induced by amyloid-β (Aβ) in mice by the rescue of TGF-β1 (Torrisi et al., 2019). Furthermore, antidepressant drugs exert immunoregulatory effects reducing the production of pro-inflammatory cytokines and stimulating the synthesis of TGF-β1 in depressed patients (Sutcigil et al., 2007).

Noteworthy, it has been shown that epigenetic mechanisms such as DNA methylation, microRNAs, and histone modifications are able to influence the development of depression (Lin and Tsai, 2019) and, with specific regard to BDNF, their altered activity can in turn affect the expression and the activity of this neurotrophic factor (Hing et al., 2018).

Several studies have demonstrated that aerobic exercise (AE) could represent a non-pharmacological strategy to improve the treatment of depression, decreasing, at the same time, the burden of somatic comorbidity of this pathology (Mura and Carta, 2013; Josefsson et al., 2014). Since the 1980s, several papers have reported on the beneficial effects played by exercise and physical activity in the treatment of depression, effects comparable to those of antidepressants (Martinsen et al., 1985; Babyak et al., 2000; Belvederi Murri et al., 2018; Lopez-Torres Hidalgo, 2019). This increased interest in this field has led to the proposal that physical exercise may serve as an alternative or integrative approach in combination with monoaminergic drugs for the treatment of MDD (Martinsen, 2008).

In the present review we will provide a general overview on the possible synergism between physical activity and antidepressants in treatment of MDD, analyzing the possible benefits of physical activity both at a neurobiological level and clinical level focusing in particular on the treatment of affective and cognitive symptoms in MDD.

## The Pathophysiology of Depression: the Role of Neurotrophic Factors and the Possible Impact of Physical Activity

MDD shows a complex pathophysiology that has been only partially elucidated in the last 10 years (Caraci et al., 2018a). Chronic stress, reduced synaptic plasticity, impairment of adult hippocampal neurogenesis, and hippocampal neurodegeneration along with the well-known dysregulation of the monoaminergic system contribute to explain the pathophysiology of MDD (Jaggar et al., 2019; Vu, 2019). Epidemiological studies support the pivotal role played by chronic stress in MDD (Pittenger and Duman, 2008); in fact, the exposure to stressful life events contributes to the development of this disease (Czeh and Lucassen, 2007). Chronic stress leads to an impaired negative feedback of glucocorticoids (GR) on the activity of HPA axis, which results in elevated cortisol levels (de Kloet et al., 2005). Excess of GR is able to induce neuronal death at hippocampal level (Yu et al., 2008) as well as dysfunctional changes in the prefrontal cortex (PFC), two regions critically involved in the cognitive symptoms of depression (Krishnan and Nestler, 2008). Stress also exerts its effects by reducing the synthesis of factors essential for neuronal homeostasis such as BDNF (Nowacka and Obuchowicz, 2013), a neurotrophin fundamental for the maintenance of dendritic spines (Vigers et al., 2012), the regulation of adult hippocampal neurogenesis (Vilar and Mira, 2016), cognitive and mood-related behavior and aging (Castren and Kojima, 2017). Reduced levels of BDNF have been connected to dendritic atrophy, neuronal apoptosis, and inhibition of neurogenesis in MDD (Nowacka and Obuchowicz, 2013). Stress decreases BDNF concentrations in hippocampus and PFC of animal models of depression (Smith et al., 1995; Duman and Monteggia, 2006; Filho et al., 2015), in line with the reduced expression of this neurotrophic factor observed at cortical, hippocampal, and peripheral level of depressed patients (Thompson Ray et al., 2011; Reinhart et al., 2015). Stress exposure also leads to an impairment of TGF-β1 signaling in different brain regions (hippocampus, cortex, and hypothalamus) (You et al., 2011; Caraci et al., 2015). This impairment has been connected to the onset of a depressive-like phenotype in mice (Torrisi et al., 2019). Lastly, a correlation between reduced TGF-β1 plasma levels, depression severity, and treatment resistance in MDD has been proved (Sutcigil et al., 2007; Caraci et al., 2018a).

In addition to HPA axis hyperactivation, immune system dysregulation and neuroinflammation play a central role in the pathophysiology of depression (Caraci et al., 2010), underlining the great impact of immune system activation on the central nervous system and in particular on the overall activity of monoaminergic systems (Caraci et al., 2018a). An increase of two well-known pro-inflammatory cytokines, called interleukin (IL)-1β and tumor necrosis factor-α (TNF-α), as well as a decrease of anti-inflammatory cytokines (e.g., IL-10, IL-4, and TGF-β1) have been observed in hippocampus and cortex of animal models of depression (You et al., 2011) and MDD patients (Farooq et al., 2017; Caruso et al., 2019).

Antidepressant drugs, such as sertraline and fluoxetine, exert immunomodulatory effects, reducing the production of pro-inflammatory cytokines and stimulating the synthesis of TGF-β1 in depressed patients (Sutcigil et al., 2007; Maes et al., 2016; Caraci et al., 2018a). Furthermore, the ability of some antidepressant drugs to induce the synthesis and the release of BDNF and TGF-β1 has been demonstrated both *in vitro* and *in vivo* studies (Caraci et al., 2010), suggesting that the long time required for BDNF restore could, at least in part, contributes to explain the therapeutic latency (2–4 weeks) of these drugs (Racagni and Popoli, 2010). Recent studies have demonstrated the rapid and long-lasting antidepressant effects of TGF-β1 as well as the key role of TGF-β1 released from microglia in mediating the antidepressant activity of (R)-ketamine (10 mg/kg) in a mouse model of depression (Zhang et al., 2020). (R)-ketamine is a novel drug under study for treatment-resistant MDD patients. Interestingly this drug rescued the expression of TGF-β1 and its receptors in the PFC and hippocampus, whereas inhibition of TGF-β1 signaling (i.e., SB431542) or neutralizing antibody of TGF-β1 blocked the antidepressant effects of (R)-ketamine, thus suggesting the essential and novel role of TGF-β1 as antidepressant.

According to the neurotrophic hypothesis of depression, which could be the impact of physical activity on the neurobiology of depression considering recent evidence in MDD patients?

**FIGURE 1:**
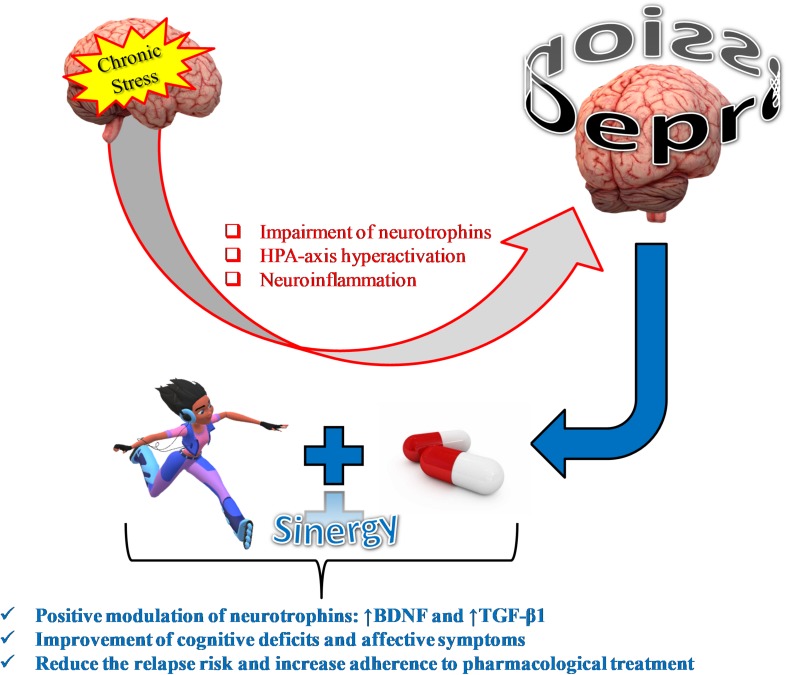
Physical activity as an add-on treatment strategy to antagonize stress-induced depression.

Physical activity as an add-on strategy to the traditional treatment of depression is able to reduce the relapse risk, increase adherence to pharmacological treatment, and promote the management of side effects with a 60–80% of success (Neumeyer-Gromen et al., 2004; Silveira et al., 2013; Figure 1).

Interestingly a recent study conducted by Murri et al. (2018), has demonstrated that physical exercise, in combination with the SSRI sertraline, reduces affective symptoms and psychomotor retardation in MDD. Furthermore, the beneficial effects of AE as an add-on strategy in the treatment of moderate to severe depression has been shown in a study carried out by Imboden et al. (2019), considering different psychological and biological variables (e.g., BDNF, HPA axis activity, cognitive symptoms) besides depression severity.

Physical activity exerts beneficial effects on pre- and postnatal brain development (Gomes da Silva and Arida, 2015), stimulates neurogenesis and synaptic plasticity by increasing BDNF synthesis and release (Walsh and Tschakovsky, 2018), and reduces HPA axis hyperactivation (Nabkasorn et al., 2006). In particular, it has been proposed, as a proof of muscle-brain crosstalk, that irisin, produced during exercise through the cleavage of fibronectin type III domain-containing protein 5 (FNDC5) membrane protein and able to cross the blood-brain barrier, induces BDNF expression at brain level, which in turn will lead to an increased hippocampal neurogenesis, and therefore to enhanced learning, memory, and mood (Pedersen, 2019). With regard to TGF-β1, the plasma concentration of this neurotrophin increases in response to exercise (1 h of treadmill running) (Heinemeier et al., 2003). In a different study enrolling healthy people and Parkinson subjects, the immunomodulatory effects of moderate intensity on plasma neurotrophins levels was investigated (Szymura et al., 2020). Szymura et al. (2020) demonstrated that after completion of the 12 weeks training program the concentration of TGF-β1 as well as of other neurothophic factors (nerve growth factor and BDNF) were found to be increased only in training groups. Furthermore, in a study considering a total of 29 athletes, the serum levels of TGF-β1 were higher in athletes with high relative Vo_2_peak (relVo_2_peak) values, a measure of the athletes’ cardiovascular fitness and aerobic endurance, compared to low relVo_2_peak (Weinhold et al., 2016). No studies have been conducted yet in MDD patients to assess whether SSRIs can synergize with AE to increase TGF-β1 signaling, although preliminary available evidence suggests the existence of common biological targets.

**FIGURE 2:**
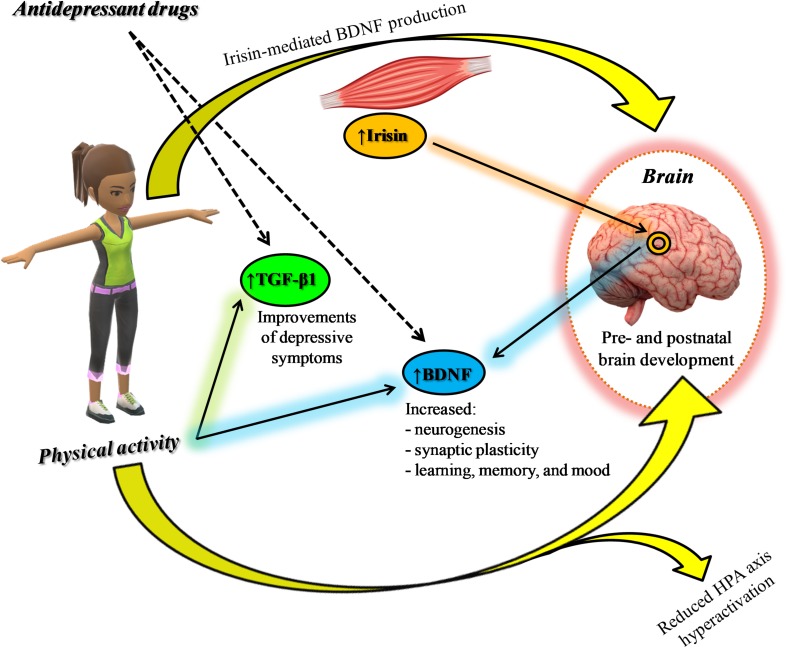
Synergic effect between physical activity and antidepressants: positive modulation of neurotrophic factors.

All together, the above mentioned evidence suggests a synergistic effect between AE and antidepressant drugs for the treatment of depression (Figure 2), reducing the cognitive deficits that compromise the working activities of MDD patients and influence their relapse risk (Albert et al., 2016). This synergism might be particularly relevant in elderly patients with late-life depression (LLD), a clinical sub-group with an increased risk to develop dementia, improving patients’ cognitive outcomes (Neviani et al., 2017).

## Impact of Physical Activity on Affective Symptoms in MDD

Apart from biological and genetic risk factors (Hammen, 2018), physical inactivity has been identified as a risk factor for the development of depression (Adamson et al., 2016; Hammen, 2018). Along this line different studies have shown that physical activity is able to provide mental health benefits in patients with severe mental illness, reducing depressive symptoms and improving social and cognitive functions (Rosenbaum et al., 2014). In a recent global systematic review and meta-analysis including 69 studies, Vancampfort et al. (2017) have examined sedentary behavior and levels of physical activity in patients with MDD or other severe mental disorders. After the analysis of the studies, it was clear as the physical activity was connected to health benefits in healthy controls while the level of activity as well as the related benefits were low in people with severe mental illnesses (Vancampfort et al., 2017). Indeed, as confirmed in different studies, regular physical activity of moderate intensity, such as walking or cycling, is enough to give significant benefits for health and plays a protective role in preventing different mental disorders (Ashdown-Franks et al., 2019); whereas lack of exercise represents a major cause of chronic diseases, including depression (Booth et al., 2012). Several studies have focused their attention on the potential benefits of physical activity to prevent the development of this disease. The “HUNT Cohort Study” investigated whether exercise provides protection against new-onset depression, the importance of both intensity and amount of physical activity and existing associations between them (Harvey et al., 2018). The results based on a healthy cohort of 33,908 adults followed for 11 years suggested that regular leisure-time exercise of any intensity provides protection against future depression development. Very recently Bennie et al. (2020) showed in a study employing a large sample (23,635) of German adults that AE is associated with a lower likelihood of depressive symptoms severity, as assessed by eight-item Patient Health Questionnaire depression scale (PHQ-8). In a case report published by Büyükturan et al. (2017), AE was able to improve physical conditions and to dramatically decrease depressive symptoms (sadness, anhedonia, reluctance to getting out of house, memory complaints) in a 76 years old female patient. Before enrollment in the study, she followed a 6-months treatment with antidepressants without getting any improvement. She followed a special 4-weeks exercise program consisting of 10 min warm-up (jogging, breathing, upper, and lower extremity active exercises), 20–25 min flexibility, balance and strengthening exercise, and 10 min cool-down exercise periods.

It has been shown that regular physical activity is able to reduce sleep disturbances (Chen et al., 2010) and improve somatic, affective, and cognitive symptoms in depressed patients, especially by enhancing the psychological health and social relationships (Babyak et al., 2000). Netz (2017), by the analysis of different randomized controlled trials in which physical exercise and pharmacologic treatment were compared, found in all studies considered, except one, that patients performing physical activity as an adjunctive treatment for depression have a significant improvement of depressive symptoms and a better clinical response after the exercise period.

An additional meta-analysis, carried out by Kvam et al. (2016), shows as different types of exercise (e.g., walking, running, cycling) could represent a viable adjunct treatment in combination with antidepressants. They demonstrate that the effects of exercise as an independent treatment were evident, with maximum efficacy showed when compared to no intervention, suggesting that physical activity may represent an alternative approach in non-responder patients.

As discussed above, a regular physical activity is also able to reduce depressive symptoms by different neurobiological mechanisms. In fact, it can increase monoaminergic neurotransmission (Stenman and Lilja, 2013), reduce cortisol levels simultaneously increasing hippocampal neurogenesis (Rethorst et al., 2009; Blake, 2012; Niwa et al., 2016), and increase β-endorphin and BDNF levels (Ernst et al., 2006). Since in the brain, neurons are a significant source of BDNF, whose synthesis occurs in regions fundamental for emotional and cognitive functions (Sasi et al., 2017), these preliminary evidence suggests that physical activity, when performed in combination with pharmacological antidepressant treatment, may improve affective symptoms in MDD patients.

## Impact of Physical Exercise on Cognitive Symptoms in MDD

MDD could be considered as the most common mental illness among elderly people with an estimated prevalence ranging from 4.6 to 9.3% (Luppa et al., 2012). Rates of MDD are by and large lower in healthy community-dwelling elderly people than in younger adult populations, in a range from 1 to 3% (Kessler et al., 2010). However, these rates can increase on the basis of increasing medical and psychiatric comorbidity as well as in relation to various social conditions (Espinoza and Kaufman, 2014). LLD, occurring in people with an age ≥60 years, is often associated to cognitive dysfunction (Taylor, 2014). Cognitive dysfunction can affect one or multiple cognitive domains such as attention, working memory, verbal fluency, visuospatial abilities, and executive function (Murrough et al., 2011). Furthermore, this clinical sub-group presents a higher risk to develop dementia, in particular Alzheimer’s disease and vascular dementia (Caraci et al., 2010; Diniz et al., 2013). LLD clinical manifestations are individual suffering, increased morbidity, premature mortality, and greater healthcare utilization (Diniz et al., 2013; Meijer et al., 2013), compromising the geriatric patients’ life quality (Morimoto et al., 2015). Moreover, LLD is a condition often accompanied by significant impairment in physical and social functioning as well as disability (Blazer, 2003; Chang et al., 2016). Longitudinal studies have shown that LLD worsens the outcomes of physical illnesses and the likelihood of frailty in elderly people (Butters et al., 2008; Vaughan et al., 2015). Research community has focused its attention on physical exercise as a potential non-pharmacological treatment to improve cognitive function in depressed elderly patients. Since 1990s, several studies have been carried out to demonstrate the efficacy of physical exercise as an intervention for clinical depression in these patients (Dupuis and Smale, 1995; Blumenthal et al., 1999). In a randomized controlled trial, Singh et al. (2001) demonstrated the effectiveness of a 20 weeks physical exercise program as a long-term treatment for clinical depression in elderly patients. The same year, in a different study, the effectiveness of a structured exercise program on specific areas of cognitive functioning (e.g., attention, concentration, executive processes, figural memory) compared to antidepressants treatment has been proved (Khatri et al., 2001). In 2012, Bridle et al. (2012) showed that structured physical exercise tailored to individual ability reduces depression severity in older people with clinically significant symptoms of depression. More recently, Heinzel et al. (2015) demonstrated that all investigated types of physical exercise, such as AE, resistance training, dancing, and alternative forms of exercise (Qi Gong and Tai Chi), may serve as a feasible and additional intervention for depression in elderly people. This preliminary evidence was strengthen by a meta-analysis of randomized controlled trials carried out by Schuch et al. (2016), suggesting that previous meta-analyses have underestimated the benefits of exercise and therefore structural physical exercise should be considered as a routine component of the management of depression in older adults.

This evidence shows how physical exercise could improve the effectiveness of pharmacological treatments in elderly depressed patients (Mura and Carta, 2013; Murri et al., 2015). An improvement in memory and executive functions that persists for up to 24 months was demonstrated in elderly depressed patients who followed an integrative approach consisting of combined physical activity and pharmacological treatment (Bragin et al., 2005). Murri et al. (2015), in a study of 24 weeks employing 121 primary care patients (>65 years) with major depression, demonstrated the synergism between the antidepressant sertraline and two different types of physical exercise in improving the outcomes related to LLD. In particular, a higher remission rate (primary outcome) was observed for the higher intensity, progressive AE plus sertraline group (81%), showing an increment of +8% (and shorter time to remission) and +36% compared to lower-intensity, non-progressive exercise plus antidepressant and sertraline alone, respectively.

Neviani et al. (2017) performed secondary analyses on data from the Safety and Efficacy of Exercise for Depression in Seniors study, a trial comparing the effectiveness of sertraline, in the absence or in the presence of progressive or non-progressive exercise. The results of 121 patients (mean age 75 years) showed improvements of Montreal Cognitive Assessment (MoCA) total scores and visuospatial/executive functions for sertraline plus progressive exercise group, showing how the addition of aerobic, progressive exercise to antidepressant drug treatment may offer significant advantages over standard treatment with regard to cognitive abilities and disability (Neviani et al., 2017).

## Conclusion

Physical activity stimulates neurogenesis and synaptic plasticity through BDNF synthesis and release, induces physiological changes in endorphine and monoamine levels, increases the plasma concentration of TGF-β1, and reduces cortisol levels; it can also act as an “anti-inflammatory factor” increasing IL-10 levels and suppressing TNF-α production, thus exerting “antidepressant-like effects”. Therefore, we can assert that physical activity modulates many mechanisms and systems involved in the pathophysiology of depression. Physical activity has also proved able to act on the core symptoms of depression, decreasing sadness, anhedonia, and sleep disturbances, improving metabolic control and cognitive functions such as attention and concentration, and also decreasing the risk of depression and dementia development. Lastly, different clinical trials have highlighted the effects of physical activity as add-on treatment for MDD patients with moderate to severe depression, underlining the existing synergism between AE and the traditional pharmacological treatment. This synergism might be particularly relevant in elderly patients with LLD, a clinical sub-group characterized by an increased risk to develop dementia.

## Author Contributions

All authors listed have made a substantial, direct and intellectual contribution to the work, and approved it for publication.

## Conflict of Interest

The authors declare that the research was conducted in the absence of any commercial or financial relationships that could be construed as a potential conflict of interest.

## Footnotes

1 https://www.who.int/health-topics/depression#tab=tab_1
